# Omega Entropy from brain functional MRI is differentially associated with Cognitive performance

**DOI:** 10.1002/alz70856_103759

**Published:** 2025-12-26

**Authors:** Norman Scheel, Jonathan M Reader, Arijit K Bhaumik, David C Zhu, Scott J. Peltier, Benjamin M. Hampstead, Bruno Giordani, Henry L Paulson, Jessica S. Damoiseaux

**Affiliations:** ^1^ Michigan State University, East Lansing, MI, USA; ^2^ Michigan Alzheimer's Disease Research Center, Ann Arbor, MI, USA; ^3^ Albert Einstein College of Medicine, Bronx, NY, USA; ^4^ University of Michigan, Ann Arbor, MI, USA; ^5^ Wayne State University, Detroit, MI, USA

## Abstract

**Background:**

Resting‐state functional MRI (rs‐fMRI) connectivity has been widely studied, but grasping the complexity of brain activity has been difficult. Dimensional complexity measures like Omega Entropy (Ω) are based on Eigenvalue (EV) spectrum analyses and can be used to estimate the complexity of rs‐fMRI brain activity. A low Ω infers stronger coherence of brain activity, while a high Ω infers less coherence. We attempt to understand the association between Ω and cognitive performance, in an older age cohort.

**Method:**

We selected a sample of participants from the Michigan Alzheimer's Disease Research Center (P30AG072931) University of Michigan Memory and Aging Project longitudinal cohort (*n* = 132, 88 female, 93 White, 37 Black/African American, ages 73 ± 6.4 years) comprised of individuals ranging from cognitively normal (*n* = 86) to mild cognitive impairment (*n* = 18) and Alzheimer's disease (*n* = 28). All participants underwent baseline rs‐fMRI and the National Alzheimer's Coordinating Center Uniform Data Set cognitive test battery, including the mental status measure, Montreal Cognitive Assessment (MoCA), and the Hopkins Verbal Learning Test (HVLT), a list‐learning memory test. Pearson correlations between cognitive scores of MoCA and HVLT, and Ω measures of the brain networks from the rrAD420 atlas were carried out. Significance was set at *p* <0.05 after corrections using the Bonferroni method.

**Result:**

HVLT total learning z‐scores were negatively correlated with Default Mode Network (DMN) Ω (*r* = ‐0.3, *p* = 0.005) and positively correlated with the limbic system Ω (*r* = 0.37, *p* = 0.009). HVLT delayed recall z‐scores were negatively correlated with DMN Ω (*r* = ‐0.25, *p* = 0.019). MoCA total scores were negatively correlated with DMN Ω (*r* = ‐0.21, *p* = 0.015).

**Conclusion:**

Higher cognitive scores in HVLT and MoCA were associated with lower DMN Ω, while higher HVLT total recall scores were associated with higher Ω in the limbic system. These results suggest more coherent brain activity in the DMN and less coherent activity in the Limbic system are associated with better cognitive performance. Our previous findings suggest Ω generally increases as we age, so especially the differential effect in the limbic system warrants further research. Overall Ω shows promise as a descriptor of cognitive states and needs to be further evaluated.

